# Enhanced cancer therapy through synergetic photodynamic/immune checkpoint blockade mediated by a liposomal conjugate comprised of porphyrin and IDO inhibitor

**DOI:** 10.7150/thno.35343

**Published:** 2019-07-29

**Authors:** Zeqian Huang, Gaofei Wei, Zishan Zeng, Yanjuan Huang, Liangfeng Huang, Yifeng Shen, Xiaoqi Sun, Congjun Xu, Chunshun Zhao

**Affiliations:** School of Pharmaceutical Sciences, Sun Yat-sen University, Guangzhou 510006, People's Republic of China

**Keywords:** protoporphyrin Ⅸ, photodynamic therapy, indoleamine-2, 3-dioxygenase, checkpoint blockade, liposome

## Abstract

Cancer metastases is still a hurdle for good prognosis and live quality of breast cancer patients. Treatment strategies that can inhibit metastatic cancer while treating primary cancer are needed to improve the therapeutic effect of breast cancer.

**Methods:** In this study, a dual functional drug conjugate comprised of protoporphyrin IX and NLG919, a potent indoleamine-2,3-dioxygenase (IDO) inhibitor, is designed to combine photodynamic therapy and immune checkpoint blockade to achieve both primary tumor and distant metastases inhibition. Liposomal delivery is applied to improve the biocompatibility and tumor accumulation of the drug conjugate (PpIX-NLG@Lipo). A series of *in vitro* and *in vivo* experiments were carried out to examine the PDT effect and IDO inhibition activity of PpIX-NLG@Lipo, and subsequently evaluate its anti-tumor capability in the bilateral 4T1 tumor-bearing mice.

**Results:** The *in vitro* and *in vivo* experiments demonstrated that PpIX-NLG@Lipo possess strong ability of ROS generation to damage cancer cells directly through PDT. Meanwhile, PpIX-NLG@ Lipo can induce immunogenic cell death to elicit the host immune system. Furthermore, PpIX-NLG@Lipo interferes the activity of IDO, which can amplify PDT-induced immune responses, leading to an increasing amount of CD8^+^ T lymphocytes infiltrated into tumor site, finally achieve both primary and distant tumor inhibition.

**Conclusion:** This work presents a novel conjugate approach to synergize photodynamic therapy and IDO blockade for enhanced cancer therapy through simultaneously inhibiting both primary and distant metastatic tumor.

## Introduction

Breast cancer is the most common cancer among women and 90 % of lethality in breast patients was caused by cancer metastases all over the world 1. The metastases are usually undetectable and remain latent for many years following primary tumor removal, which may lead to a poor prognosis and low five-year survivals rate of breast cancer patients 2. Therefore, treatment strategies that can inhibit metastatic cancer while treating primary breast cancer are needed to improve the prognosis and live quality of breast cancer patients.

Photodynamic therapy (PDT), which utilizes photosensitizers, oxygen and light with specific wavelength to generate cytotoxic reactive oxygen species (ROS), has attracted substantial research interest as an emerging treatment strategy for cancer therapy over the past decade 3, 4. With significant advantage of spatiotemporal controllability, minimal invasiveness and irreversible destruction, PDT has been approved for clinical application by Food and Drug Administration of the United Stated for the treatment of various of solid tumors 5. Very recently, many studies indicated that PDT could not only damage cancer cells directly to inhibit primary tumor, but also control distant metastases by initiating antitumor immune responses 6-9. It has been verified that PDT can cause immunogenic cell death (ICD) during damaging cancer cells to release tumor-associated antigens, which can stimulate the host immune system, subsequently lead to the proliferation and activation of CD8^+^ T lymphocytes (CD8^+^ T cells) 10-13. Nevertheless, the immune response induced by PDT could be severely impaired by immunosuppressive tumor microenvironment, which had formed during the development of tumors to evade host immune surveillance 14-16.

Recently, checkpoint blockade inhibitors, the modulator of immunosuppressive tumor microenvironment, have been applied to amplify the PDT-mediated immune response to improve the anti-tumor outcome of PDT. 6-8, 17-19. Checkpoint blockade as a promising strategy of cancer immunotherapy has attracted tremendous interests in recent years 20-23. Among the immune checkpoint, indoleamine-2,3-dioxygenase (IDO) is such a special checkpoint for its inhibitors are some small-molecule drug 24. IDO, highly expressed in many types of solid tumors, is an immunoregulatory enzyme that catalyzes the oxidative metabolism of tryptophan (Trp) to kynurenine (Kyn) 25. The consequential unavailability of tryptophan and accumulation of kynurenine in tumor microenvironment could blunt the proliferation of T cells, promote the generation and activation of T regulatory cells, helpfully forming the immunosuppressive microenvironment in tumor sites 26.Therefore, IDO inhibitors, which can relieve immunosuppressive tumor microenvironment and elicit the host immune system, exhibit promising application for checkpoint blockade 27. Among the IDO inhibitors, NLG919 is a novel candidate drug with potential immunomodulating and antineoplastic activities, whose values of inhibition constant (*K_i_*) and half maximal effective concentration (EC_50_) of 7 nM and 75 nM respectively 28.

**Scheme 1:**
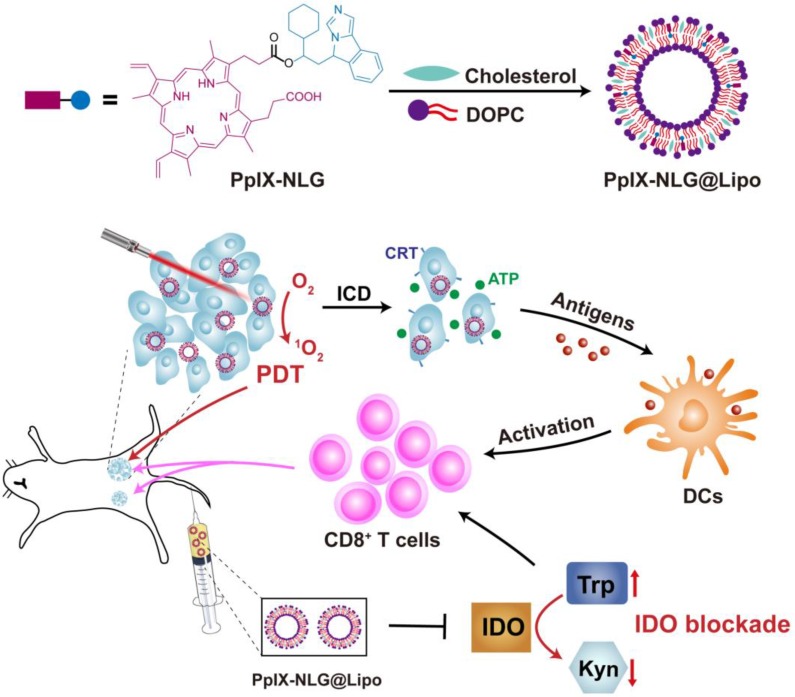
Schematic illustration of PpIX-NLG@Lipo for combined PDT and IDO blockade. Firstly, PDT mediated by PpIX-NLG@Lipo under laser irradiation can damage primary cancer cells directly. In the meanwhile, the combination of PDT-mediated antigens presentation and IDO blockade can induce more CD8^+^ T cells proliferation and infiltration into tumor sites, resulting in both inhibition of primary tumors and distant tumors.

From the above, the synergetic application of PDT and IDO blockade may be a better treatment strategy to improve the therapeutic effect on the inhibition of both primary and distant tumor 6. Moreover, keep the ratio and pharmacokinetic properties of photosensitizer and IDO inhibitor consistently may contribute to the preferable synergetic therapeutic efficacy of antitumor. Thus, in order to achieve the dual function of PDT and IDO blockade, the drug conjugate PpⅨ-NLG was synthesized through linking IDO inhibitor NLG919 to photosensitizer PpIX via ester bond. The liposome system was selected as a drug carrier for PpIX-NLG (termed as PpIX-NLG@Lipo) to improve its biocompatibility as well as tumor accumulation. We hypothesized that the nanoscale PpIX-NLG@Lipo can preferentially accumulate into tumor site after intravenous injection and generate ROS under light irradiation to damage cells of primary tumors directly, in the meanwhile cause immunogenic cell death (ICD) to enhance the immunogenic of tumors. Meanwhile, PpIX-NLG@Lipo can interfere IDO activity to modulate tryptophan/kynurenine metabolism, finally reverse the immunosuppressive tumor microenvironment. The combination of PDT and IDO blockade lead to amplified CD8^+^ T cells proliferation and infiltration, resulting in both inhibition of primary, treated tumors but also rejection of distant, untreated tumors (**Scheme 1**). As a result, this work presents a novel conjugate approach to synergize photodynamic therapy and IDO blockade for enhanced cancer therapy through simultaneously inhibiting both primary and distant metastatic tumor.

## Materials and Methods

### Materials, cells and animals

Protoporphyrin IX (PpIX) was purchased from Howei Pharm (Guangzhou, China), The small molecule IDO inhibitor NLG919 was purchased from SuperLan Chemical (Shanghai, China). L-Kynurenine Hydrate (>98%) was purchased from TCI (Tokyo, Japan). 9,10-anthracenediyl-bis(methylene)dimalonic acid (ABDA), fluorescein diacetate (FDA), L-Tryptophan (>99%) were purchased from Aladdin Industrial Corporation (Shanghai, China). 2′,7′-dichlorofluorescein diacetate (DCFH-DA), 3-(4.5-dimethyl-thiazol-2- yl)-2.5-diphenyl tetrazolium bromide (MTT), 1,3- Diphenylisobenzofuran (DPBF) were purchased from Sigma-Aldrich (St. Louis, USA). Propidium Iodide (PI) was purchased from Beyotime (Shanghai, China). 1,2-dioleoylsn-glycero-3-phosphocholine (DOPC) and cholesterol were purchased from Avanti Polar Lipids, Inc. (Alabaster, USA). Roswell Park Memorial Institute (RPMI) 1640 medium, Dulbecco's Modified Eagle's Medium (DMEM), pancreatic enzymes, fetal bovine serum (FBS) and IFN-γ Recombinant Human Protein were purchased from Gibco. IDO1 antibody (Proteintech Group, USA) and anti-mouse IDO Antibody (biolegend, Japan) was purchased for Western blot and Immunohistochemistry (IHC), respectively. Alexa Fluor 488-Calreticulin Antibody was purchased from Novus Biologicals (Colorado, USA). Annexin V/PI Apoptosis Detection Kit, CD3-FITC antibody, CD8α-PE antibody were purchased from eBiosciences (Hatfield, UK).

Human breast cancer cells MCF-7, murine breast cancer cells 4T1 and Hela cells were provided by Laboratory Animal Center of Sun Yat-sen University (Guangzhou, China). MCF-7 was cultured in RPMI 1640 supplemented with 10% FBS under the atmosphere of 5% CO_2_ at 37 °C, 4T1 and Hela cells were cultured in DMEM supplemented with 10% FBS under the atmosphere of 5% CO_2_ at 37 °C.

BALB/c female mice (5-6 weeks old) were provided by the Laboratory Animal Center of Sun Yat-sen University (Guangzhou, China) and kept under specific pathogen free condition with free access to standard food and water. All experimental procedures were approved and supervised by the Institutional Animal Care and Use Committee of Sun Yat-sen University. Sprague-Dawley rats (200 ± 20 g) were supplied by the Laboratory Animal Center of Sun Yat-sen University (Guangzhou, China).

### Synthesis of PpIX-NLG

To a solution of PpIX (50.0 mg, 0.0888 mmol) and NLG919 (25.1 mg, 0.0888 mmol) in dry DMF (5mL), EDCI (25.5 mg, 0.1332 mmol) and DMAP (1.6 mg, 0.0133 mmol) were added and stirring for 5 h at 60 ℃. Subsequently, the mixture was filtered and the filtrates were collected. The obtained filtrates were concentrated and purified by a silica gel column chromatography using petroleum ether/ethyl acetate (4:1) as eluent to afford a red solid (48.3 mg, 65.8%). HRMS(ESI): m/z calculated for C52H55O4N6 [M+H]^+^: 827.42793, Found 827.42854. FT-IR: 3309.30 cm^-1^ for -OH, 1724.08 cm^-1^ for -CO-.

### Preparation and characterization of liposomes

PpIX-NLG loaded liposome (PpIX-NLG@Lipo) was prepared through the thin-film dispersion method. Briefly, DOPC, cholesterol and PpIX-NLG at a mole ratio of 8.5: 3.5: 1 were dissolved in tetrahydrofuran (THF), then evaporating the THF solvent until forming a lipid thin film. Subsequently, the obtained thin film was hydrated in phosphate buffered saline (PBS) at a temperature of 40 °C, and sonicated using an ultrasonic cleaner for 10 min to get the nanoscale PpIX-NLG loaded liposome (PpIX- NLG@Lipo). The NLG919 loaded liposome (NLG@ Lipo) and PpIX loaded liposome (PpIX@Lipo) was prepared through the same method.

The DLS and UV-visible absorption spectra of PpIX-NLG@Lipo, PpIX@Lipo and NLG@Lipo in aqueous solution were measured by a Malvern Zetasizer Nano ZS90 (Malvern Instruments, Malvern, UK) and UV-vis spectrophotometer (TECHCOMP, UV2600 spectrophotometer), respectively. Transmission electron microscopy (TEM) (JEM-1400, JEOL, Japan) was used to observe the morphology of PpIX-NLG@Lipo. The loading capacity of PpIX-NLG (or PpIX) and NLG919 was quantified with fluorospectrophotometer (Fluoromax-4, Horiba, Japan) and HPLC, respectively.

### ROS generation detection

The detection of ROS generated from free drug was performed by using DPBF as a ROS indicator. Briefly, the DMSO solution of PpIX or PpIX-NLG (the concentration of PpIX or PpIX-NLG were 5 μM) containing 62.5 μM DPBF were irradiated by LED light (630 nm, 20 mW/cm^2^) at the predetermined time points. After irradiation, the absorbance of DPBF at 416 nm was measured by UV-vis spectrophotometer to determine the ROS generation.

ROS generation of PpIX-NLG@Lipo in aqueous solution was detected by using ABDA as a water-soluble ROS indicator. Firstly, ABDA (final concentration was 80 μM) was mixed with equal volume of PpIX@Lipo or PpIX-NLG@Lipo at a final concentration of 5 μM, respectively. Then the mixture solution was exposed to laser irradiation (630 nm, 50 mW/cm^2^) at the predetermined time points, and the solution absorbance curve was recorded by a UV-vis spectrophotometer after irradiation. Subsequently, the decrease of ABDA absorbance at 401 nm was used to evaluate the ROS production.

The indicator SOSG was applied to detect the generation of singlet oxygen of PpIX-NLG@Lipo under light irradiation. Briefly, 100 μg of SOSG was diluted in 165 μL of methanol to achieve the 1 mM SOSG stock for preparation. A 5 μL of SOSG stock was added into a 5 mL sample with a certain concentration. The samples were then irradiated by LED light (630 nm, 20 mW/cm^2^) for different times, respectively, and the fluorescence change was recorded with an excitation wavelength of 504 nm and an emission wavelength of 525 nm.

Intracellular ROS generated from PpIX-NLG@ Lipo under light irradiation was determined by flow cytometry (FCM) and confocal laser scanning microscopy (CLSM). For FCM analysis, MCF-7 cells or 4T1 cells seeded in 6-wells plates (1×10^5^ cells per well) were incubated with PpIX@Lipo, NLG@Lipo and PpIX-NLG@Lipo for 24 h. Afterward, cells were incubated with fresh medium containing DCFH-DA for 30 min, followed by LED light irradiation (630 nm, 20 mW/cm^2^) for 10 min. Then, treated cells were harvested and analyzed by flow cytometry system (Guava EasyCyte 6-2L, Merck Millipore). For CLSM imaging, 4T1 cells were seeded on glass coverslips in 12-well plates at a density of 5×10^4^ cells per well and incubated with PpIX@Lipo, NLG@Lipo and PpIX-NLG@Lipo (1.25 μM) for 24 h. Then, cells were incubated with fresh medium containing DCFH-DA for 30 min, followed by LED light irradiation (630 nm, 20 mW/cm^2^) for 10 min. After washed with PBS for three times, fixed with 4% paraformaldehyde, stained with DAPI, cells were observed using CLSM (FV3000, Olympus, Japan).

### *In vitro* PDT efficacy

MTT assay was performed to evaluate the PDT efficacy and phototoxicity of PpIX-NLG@Lipo *in vitro*. MCF-7 cells (1×10^4^ cells per well) or 4T1 cells (3×10^3^ cells per well) were seeded in 96-well plates and incubated with PpIX@Lipo, NLG@Lipo or PpIX- NLG@Lipo. After being incubated for 24 h, cells were exposed to LED light irradiation (630 nm, 20 mW/cm^2^) for 10 min or without irradiation, followed by incubated for another 24 h. Subsequently, MTT solution was added and incubated for another 4 h, then the culture medium was replaced with DMSO. The optical density (OD) of each well was measured at 490 nm with a microplate reader (ELX800, Bio-Tek, USA). The relative cell viability was calculated as follows: viability = (OD sample/OD control) × 100%, the cells without any treatments as a control.

The live/dead cell staining assay was also applied to visualize the PDT outcome of PpIX-NLG@Lipo with light irradiation in 4T1 cells. Briefly, 4T1 cells seeded in 12-well plates at a density of 5×10^4^ cells per well were incubated with NLG@Lipo, PpIX@Lipo and PpIX-NLG@Lipo at concentration of 0.625 μM and 1.25 μM for 24 h, following by irradiated with LED light (630 nm, 20 mW/cm^2^) irradiation for 10 min or not. After further incubation for another 24 h, cells were stained with fluorescein diacetate (FDA) and PI for 10 min, washed with PBS for three times, and subsequently observed and photographed by Inverted Fluorescent Microscope (IX73, Olympus, Japan).

Cell apoptosis analysis of PpIX-NLG@Lipo was measured by using Annexin V Apoptosis Detection Kit. Briefly, 4T1 cells (1×10^5^ cells per well) seeded into a 6-well plate were incubated with PpIX@Lipo, NLG@Lipo or PpIX-NLG@Lipo at concentration of 1.25 μM and 2.5 μM for 24 h, and subsequently irradiated with LED light (630 nm, 20 mW/cm^2^, 10 min) or not. Following incubation of another 4 h, cells were harvested, stained with Annexin V-FITC and PI according to the manufacturer's instructions, and finally analyzed by FCM.

### CRT exposure and ATP secretion assay

*In vitro* CRT exposure induced by PpIX-NLG@ Lipo were evaluated by CLSM and FCM. Briefly, 4T1 cells seeded on glass coverslips were incubated with PpIX@Lipo, NLG@Lipo and PpIX-NLG@Lipo (1.25 μM) for 24 h. Then, cells were irradiated with LED light (630 nm) at 20 mW/cm^2^ for 10 min. Following further incubation of 2 h, cells were washed with PBS three times, incubated with Alexa Fluor 488-CRT antibody for 2 h, stained with DAPI, and then observed under CLSM using 405 nm and 488 nm lasers for visualizing nuclei and CRT expression on the cell membrane, respectively. For FCM analysis, 4T1 cells seeded in 6-wells plates (1×10^5^ cells per well) were incubated with PpIX@Lipo, NLG@Lipo and PpIX-NLG@Lipo (1.25 μM) for 24 h. Afterward, cells were irradiated with LED light (630 nm) at 20 mW/cm^2^ for 10 min. Following further incubation of 2 h, the treated cells were harvested, washed twice with ice-cold PBS, incubated with Alexa Fluor 488-CRT antibody for 2 h, and finally analyzed by flow cytometry system (Guava EasyCyte 6-2L, Merck Millipore).

For ATP secretion assay, 4T1 cells (1 × 10^5^ per well) were seeded into 6-well plates and incubated with PpIX@Lipo, NLG@Lipo and PpIX-NLG@Lipo (1.25 μM) for 24 h, following by irradiated or not with LED light (630 nm, 20 mW/cm^2^, 10 min). After further incubation for 24 h, the supernatant of each well was carefully collected and dying tumor cells were removed by centrifugation and supernatants were isolated for further detection of the extracellular ATP secretion by using a luciferin-based ATP Assay kit.

### Cell-based IDO enzymatic activity

Cell-based IDO enzymatic activity assay was performed to measure the IDO inhibitory effect of NLG@Lipo and PpIX-NLG@Lipo. Hela cells were seeded in a 96-well plate with a density of 5×10^3^ cells per well, then 50 ng/mL (final concentration) of Recombinant human IFN-γ was added to each well with the aim to stimulate IDO express 27. In the meantime, various concentration of NLG@Lipo or PpIX-NLG@Lipo ranging from 0.1 μM to 20 μM were added to the cells. After incubation of 48 h, 150 μL of the supernatants per well were transferred to a new 96-well plate. For colorimetric assay, the 150 μL of the supernatants were mixed with 75 μL of 30% trichloroacetic acid, and the mixture was incubated in 50 ℃ for 30 min. After centrifugation at 5000 rpm for 8 min, 80 μL of supernatants were transferred to a new 96-well plate and following mixed with equal volume of Ehrlich reagent (2% p-dimethylamino-benzaldehyde in glacial acetic acid). The final reaction product was measured at 490 nm by a microplate reader. For HPLC detection, the 150 μL supernatants of per well were directly analyzed by HPLC for tryptophan and kynurenine measurement. The HITACHI Chromaster HPLC system (HITACHI, Japan) and a Diamonsil C18 column (4.6 mm × 250 mm, 5 μm, DiKMA, Beijing, China) was applied. Acetonitrile (A) and 15 mM sodium acetate solution containing 0.02% acetic acid (B) was used as the mobile phase (A: B=8: 92, v/v) with the flow rate of 1.0 mL/min when the column temperature was set at 25℃. The UV spectra were recorded in the range from 200 nm to 800 nm, and 218 nm and 225 nm was set up for quantification of Trp and Kyn, respectively.

MTT assay was taken to evaluate the cell viability of Hela cells during the IDO enzymatic activity assay. Briefly, after transferring 150 μL of the supernatants per well to a new 96-well plate mentioned above, 150 μL of fresh medium containing MTT was added to the cells and incubated for another 4 h. Subsequently, the culture medium was removed and replaced with DMSO. The OD was measured at 490 nm with a microplate reader. The relative cell viability was calculated as follows: viability = (OD sample/OD control) × 100%, the cells without any treatment as a control.

Western-blot analysis was employed to examine the expression of IDO during the IDO enzymatic activity assay. Hela cells seeded in a 6-well plate were incubated with 50 ng/mL of Recombinant human IFN-γ and NLG@Lipo or PpIX-NLG@Lipo for 48 h. Then, the cells were harvested, lysed and centrifuged to collect proteins. The obtained protein samples were analyzed for IDO expression through western blotting method by using tubulin as an internal control protein.

### Bilateral tumor xenograft model and *in vivo* tumor imaging

The bilateral 4T1 mouse breast cancer model was established for the following *in vivo* evaluation. BALB/c female mice (5-6 weeks old) were subcutaneously injected with 1×10^6^ 4T1 cells in the right flank and 4×10^5^ 4T1 cells in the left flank to construct the bilateral 4T1 mouse breast cancer model.

For *in vivo* distribution experiment, after the tumor volume reaching about 200 mm^3^, bilateral 4T1 tumor-bearing mice (n=3) were i.v. injected with PpIX-NLG@Lipo at a PpIX-NLG dose of 6 μmol kg^-1^. Mice were anesthetized by 4% (w/v) chloral hydrate and imaged at time point of 2, 8, 24 h respectively via small animal imaging system (Night OWL LB983, Berthold, Germany, excitation: 630 nm, emission: 680 nm). Twenty-four hours after injection, the mice were sacrificed and the heart, liver, spleen, lung, kidney, tumor were removed. Subsequently, the excised organs were imaged via small imaging system at the same condition.

### *In vivo* pharmacokinetics study

Sprague-Dawley rats weighing 200 ± 20 g were randomly divided into 2 groups (n = 3 for each group). Five hundred microliter of PpIX-NLG (which was dissolved in saline containing 3% DMSO and 3% tween-80) and PpIX-NLG@Lipo was injected into the tail vein at dose of 2.5 μmol/kg. At the predetermined time points (10 min, 30 min, 1 h, 2 h, 4 h, 6 h, 8 h, 12 h, and 24 h), the blood samples were collected from the tail vein in heparinized tubes. Blood was centrifuged at 3000 rpm for 5 min to obtain the plasma, which was diluted with DMSO before analyses of PpIX-NLG contents by using Fluor spectrophotometer (Fluoromax-4, Horiba, Japan).

### *In vivo* IDO enzyme activity

Firstly, the expression of IDO in tumor tissue of the bilateral 4T1 tumor-bearing mice were detected by immunohistochemistry (IHC). When the primary tumor reached about 100 mm^3^, the bilateral 4T1 tumor-bearing mice (n=3) were sacrificed and the tumor were dissected and sectioned for immunohistochemistry staining by IDO1 antibody to indicate the expression of IDO in tumor site.

The Kyn and Trp ratios in plasma in 4T1 tumor-bearing mice treated with PpIX-NLG@Lipo, as an indication of *in vivo* IDO enzyme activity, were examined by HPLC. After the primary tumor reached about 100 mm^3^, the bilateral 4T1 tumor-bearing mice (n=4) were treated with saline, NLG@Lipo, PpIX@Lipo and PpIX-NLG@Lipo (6 μmol NLG or PpIX per kg) via intravenous injection once every day for total three times. One day after the last treatment, the plasma sample of each mouse was harvested by using heparin as anticoagulant. Then, plasma samples were mixed with methanol for protein precipitation (plasma: methanol, 1:3, v/v), centrifuged at 12000 rpm for 20 min. Subsequently, the obtained supernatant was evaporated by using the sample concentrator and the redissolved solution was collected for HPLC quantification of Kyn and Trp. The HPLC condition was the same as what mentioned above.

### *In vivo* antitumor effect and mechanisms analysis

When the primary tumor reached about 80~100mm^3^, the bilateral 4T1 tumor-bearing mice were randomly divided into seven groups (n=6): saline; saline with light irradiation; NLG@Lipo; PpIX @Lipo; PpIX@Lipo with light irradiation; PpIX-NLG@ Lipo; PpIX-NLG@Lipo with light irradiation. Liposomes were i.v. injected to animals at a PpIX or NLG dose of 6 μmol kg^-1^ every two days for a total of three injections. After 24 h post-injection, mice were anesthetized by 4% (w/v) chloral hydrate and the primary tumors were irradiated with 630 nm laser (300 mW/cm^2^) for 10 min. The body weight and the primary and distant tumor size of each groups were monitored every two days. The tumor volume was calculated with the following formula: V = (tumor length) × (tumor width)^2^/2. After the last measurement at day 21, all the mice were sacrificed, and the tumor were removed and photographed aiming to show the therapeutic effect of each groups directly.

To evaluate the immune response of PpIX-NLG@Lipo in the bilateral 4T1 tumor-bearing mice, the infiltrated CD8^+^ T cells into tumor site were observed by IHC and FCM. The day after the last laser irradiation, the mice were sacrificed, and the tumors were removed. For FCM analysis, tumors were destroyed by mechanical method and the resulting tumor solution was isolated by passing 200 μm and 70 μm filter to get the single tumor cell suspension. The cell suspension was stained with CD3-FITC and CD8-PE antibodies at 4℃ for 30 min, subsequently detected by FCM to quantify the CD8^+^ T cells in tumor site. For IHC analysis, tumors were dissected and sectioned for staining by CD8 antibody to indicate the infiltration of CD8^+^ T cells in tumor site.

### Safety evaluation

Blood biochemical analysis and histopathological observation of the vital organs (heart, liver, spleen, lung, kidney) were taken to test the safety of PpIX-NLG@Lipo *in vivo* application. Tumor-bearing mice were randomly divided into two groups (n=5), which was intravenously injected with saline or PpIX-NLG@Lipo (the dosage of PpIX-NLG was 6 μmol kg^-1^), respectively. The day after injection, plasma sample of each mouse was collected for blood biochemical analysis. Alanine aminotransferase (ALT) and aspartate aminotransferase (AST) were measured to evaluate the liver function, while blood urea nitrogen (BUN) and creatinine (CREA) were examined to identify renal function. The measurement of ALT and AST, BUN and CREA were processed by Laboratory Animal Center of Sun Yat-sen University (Guangzhou, China). After finishing collecting plasma samples, the vital organs of each mouse were removed, fixed with 4% paraformaldehyde, embedded in paraffin and sectioned, finally stained with H&E according to the manufacturer's instructions. The stained section was observed and photographed by Inverted Fluorescent Microscope to determine their histopathological changes.

### Statistical analysis

Data were showed as mean ± S.D. All the statistical analysis was performed using SPSS Statistics 13.0 software. One-way analysis of variance was used to determine the significance of the difference. The differences were considered significant for *p < 0.05 and very significant for **p < 0.01 or ***p < 0.001.

## Results and Discussion

### Preparation and characterization of liposomes

**Figure 1:**
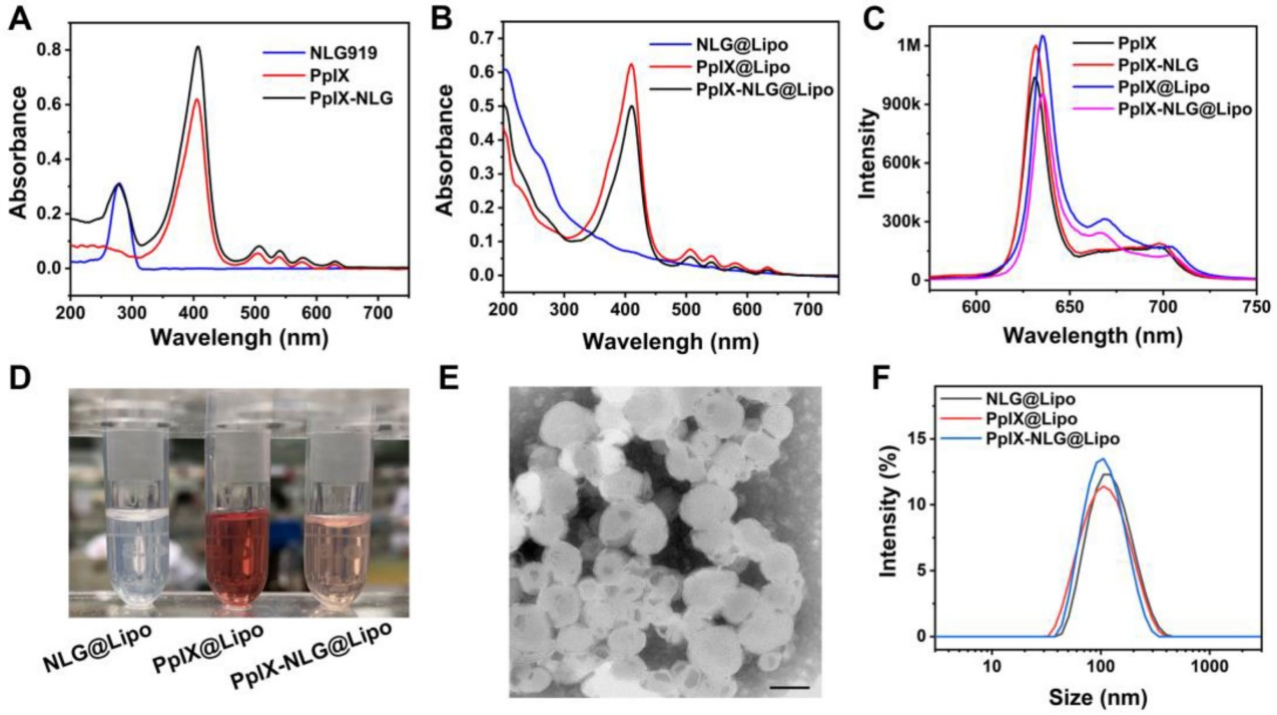
(A) UV-vis spectrum of NLG919, PpIX, and PpIX-NLG in DMSO. (B) UV-vis spectrum of NLG@Lipo, PpIX@Lipo, and PpIX-NLG@Lipo in aqueous solution. (C) Fluorescence emission spectrum of PpIX and PpIX-NLG in DMSO, PpIX@Lipo and PpIX-NLG@Lipo in aqueous solution, respectively. (D) Representative photographs of NLG@Lipo, PpIX@Lipo, and PpIX-NLG@Lipo. (E) Representative TEM image of PpIX-NLG@Lipo (scale bar =100 nm). (F) Size distribution of NLG@Lipo, PpIX@Lipo, and PpIX-NLG@Lipo in aqueous solution determined by DLS.

In order to achieve the dual function of PDT and IDO blockade, the drug conjugate PpⅨ-NLG was synthesized through linking IDO inhibitor NLG919 to photosensitizer PpIX via ester bond (**Scheme S1**). High resolution mass spectra (HRMS) (**Figure S1**) and FTIR (**Figure S2**) were shown the exact mass and characteristic functional group of PpIX-NLG respectively, indicating that PpIX-NLG was successfully synthesized. Moreover, the successful synthesis of PpIX-NLG could be also confirmed by the UV-Vis absorption spectra (**Figure 1A**), in which PpIX-NLG exhibited both absorption at 278 nm and 410 nm, which was the characteristic band of NLG919 and PpIX, respectively.

With the aim to solve the problem of low solubility of PpIX-NLG and improve its biocompatibility and tumor accumulation, the liposome drug delivery system was applied. PpIX-NLG was encapsulated into liposome (defined as PpIX-NLG@Lipo) by mixing DOPC, cholesterol and PpIX-NLG at a specific molar ratio according to thin-film dispersion method 29. As shown in **Figure 1B**, PpIX-NLG@Lipo exhibited similar characteristic absorption peak as free PpIX-NLG in UV-Vis spectroscopy, suggesting the successful loading of PpIX-NLG into liposome. In the fluorescence emission spectra (**Figure 1C**), PpIX-NLG@Lipo existed a strong fluorescence in aqueous solution at 635 nm, which was similar with that of free PpIX-NLG in DMSO, indicating that the photosensitive properties of PpIX-NLG did not impair after encapsulated into liposome. As shown in transmission electron microscopy (TEM), the as-prepared PpIX-NLG@Lipo showed typical lipid layer structure and uniform sphere-like morphology with a mean diameter of 100 nm (**Figure 1E**). The encapsulation efficiency and loading efficiency of PpIX-NLG was calculated to be 87.6% ± 0.23% and 4.25% ± 0.13%, respectively. NLG919 loaded liposome (noted as NLG@Lipo) and PpIX loaded liposome (defined as PpIX@Lipo) were prepared through the same method, respectively (**Figure 1D**). The hydrodynamic diameter of PpIX-NLG@Lipo was 98 nm measured by dynamic laser scattering (DLS), and that of NLG@Lipo and PpIX@Lipo were 100 nm and 102 nm, respectively (**Figure 1F**). The particle size changes of the PpIX-NLG@Lipo in the presence of PBS solution containing 10% FBS were monitored to evaluate its stability in physiological conditions. As shown in **Figure S3**, the particle sizes exhibited little change, suggesting the physical stability of PpIX-NLG@Lipo in physiological conditions.

### ROS generation detection

**Figure 2:**
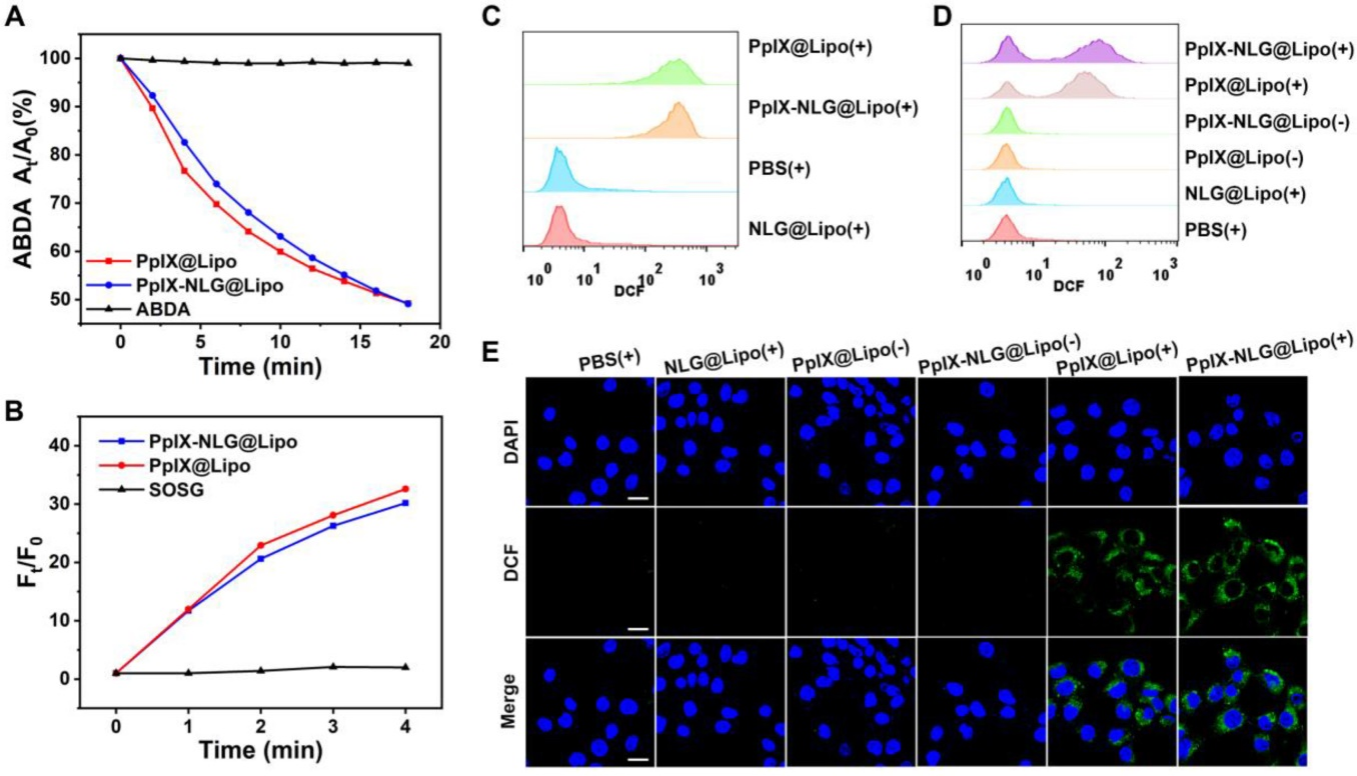
Detection of ROS generation of PpIX-NLG@Lipo. (A) ROS generation of PpIX@Lipo and PpIX-NLG@Lipo in aqueous solution with laser irradiation (630 nm, 50 mW/cm^2^) using ABDA as an ROS indicator. (B) ROS generation of PpIX@Lipo and PpIX-NLG@Lipo in aqueous solution with LED light irradiation (630 nm, 20 mW/cm^2^) using SOSG as a single oxygen indicator. Intracellular ROS generation quantified by FCM of MCF-7 cells (C) or 4T1 cells (D) incubated with PpIX-NLG@Lipo after LED light irradiation (630 nm, 20 mW/cm^2^, 10 min). (E) Intracellular ROS generation of 4T1 cells observed by CLSM (scale = 20 μm). (+) and (-) respectively refer to with or without irradiation.

The ability of ROS generation from photosensitizer is important for its efficiency of PDT treatment. Thus, we first measured the ROS-generating capability of free drug conjugate PpIX-NLG by using DPBF as an ROS indicator, which can capture and react with ROS, resulting in the decrease of its characteristic absorption peak at 416 nm 30. As exhibited in **Figure S4**, PpIX and PpIX-NLG exhibited sharp decrease of absorbance during 5 min with LED light irradiation (630 nm, 20 mW/cm^2^), while there was no obvious change of absorbance of the DPBF solution, demonstrating that both PpIX and PpIX- NLG showed a strong ability to generate ROS with light irradiation. Subsequently, the ROS- generating ability of PpIX-NLG@Lipo was detected by using ABDA as a water-soluble ROS probe. The decrement of absorbance at 401 nm, the characteristic absorption peak of ABDA, was measured to indicate the generation of ROS 31. In **Figure 2A** and **Figure S5**, PpIX-NLG@Lipo and PpIX@Lipo exhibited obvious decrease of absorbance under laser irradiation (630 nm, 50 mW/cm^2^), which suggested that PpIX-NLG@ Lipo or PpIX@Lipo could generate ROS under irradiation in the water aqueous solution. Moreover, SOSG was employed as a single oxygen indicator detect the single oxygen generated from PpIX-NLG@ Lipo in the aqueous solution, as shown in **Figure 2B**, whose result was consistent to the measurement of ABDA.

Then, intracellular ROS generation of PpIX-NLG @Lipo under light irradiation was observed by using DCFH-DA, a non-fluorescent ROS indicator, which can be hydrolyzed by intracellular esterase and further oxidized to strong fluorescent product DCF when ROS exists 30. With LED light irradiation for 10 min, the MCF-7 cells (**Figure 2C**) or 4T1 cells (**Figure 2D**) treated with PpIX@Lipo and PpIX-NLG@ Lipo displayed strong DCF fluorescence, while those treated with NLG@Lipo or PBS showed little fluorescence. The generation of ROS induced by PpIX-NLG@Lipo was observed visually by CLSM, as shown in **Figure 2E**, the obviously green fluorescence of DCF was occurred in the group of PpIX-NLG@Lipo after 10 min of light irradiation, while inappreciable DCF fluorescence was occurred in those cells without irradiation. Thus, the results discussed above demonstrated that PpIX-NLG@Lipo showed good capability of ROS production both extracellularly and intracellularly.

### *In vitro* PDT efficacy and apoptosis analysis

**Figure 3:**
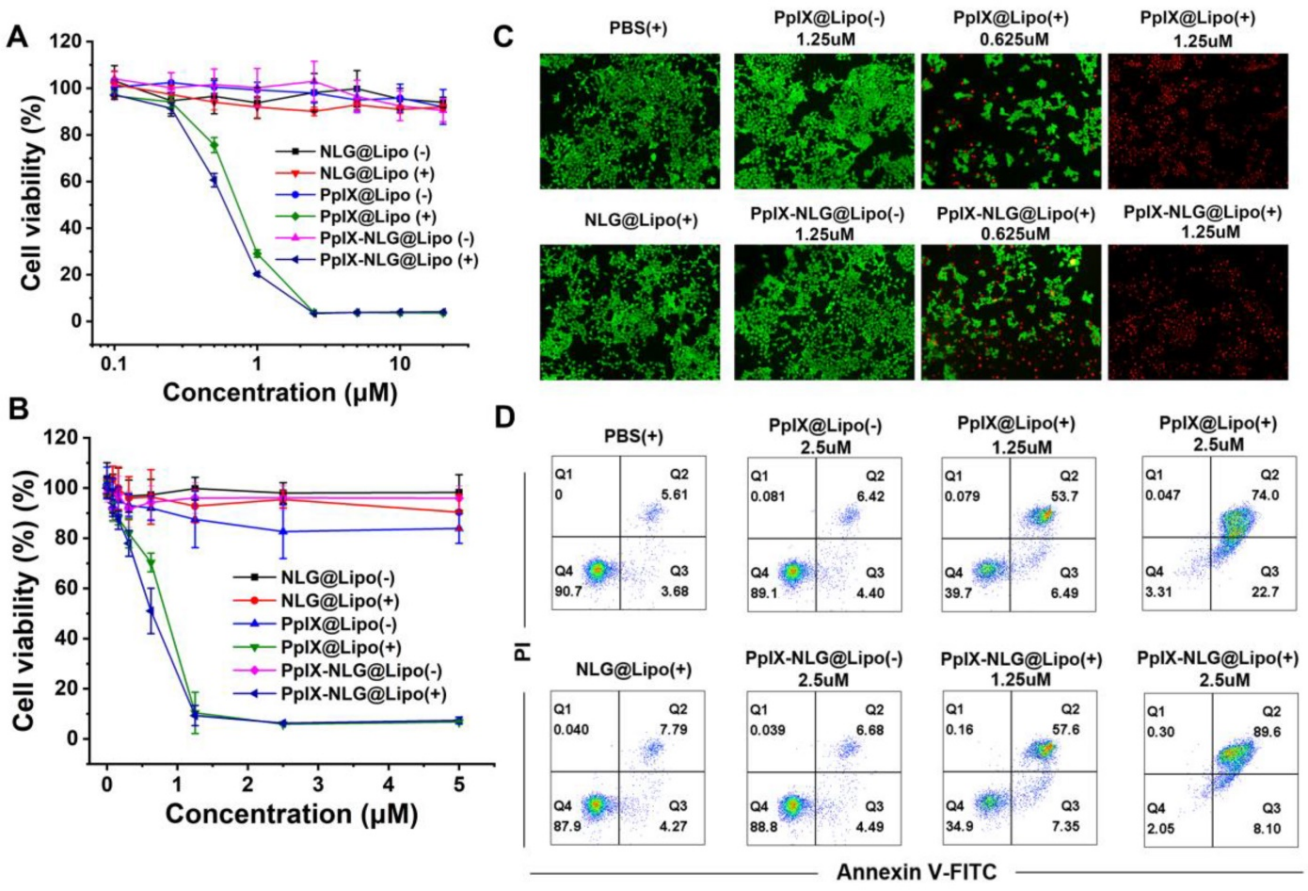
*In vitro* PDT efficacy study. The cell viability of MCF-7 cells (A) or 4T1 cells (B) with or without irradiation (630 nm, 20 mW/cm^2^, 10 min) after incubation with various concentration of NLG@Lipo, PpIX@Lipo or PpIX-NLG@Lipo, respectively. (C) The live/dead staining of 4T1 cells 24 h further incubation after irradiated or not, in which red fluorescence and green fluorescence represent dead and live cells, respectively (Scale bar = 200 μm). (D) Annexin V/PI analysis of 4T1 cells 4 h further incubation after irradiated or not after treating with PBS, NLG@Lipo, PpIX@Lipo or PpIX-NLG@Lipo. The quadrants from lower left to upper right (counter clockwise) represent healthy, early apoptotic, late apoptotic/necrotic cells, respectively. The percentage of cells in each quadrant was shown in each graph. (+) and (-) respectively refer to with or without irradiation.

After confirming the ROS generation of PpIX- NLG@Lipo in extracellularly and intracellularly, then the *in vitro* PDT efficacy and phototoxicity of PpIX-NLG@Lipo was tested by MTT assay and live/dead cell staining experiment. As **Figure 3A** and **Figure 3B** displayed, PpIX-NLG@Lipo and PpIX@ Lipo showed significant cytotoxicity under the LED light irradiation (630 nm, 20 mW/cm^2^, 10 min) on MCF-7 cells as well as 4T1 cells, while a little bit enhanced cytotoxicity of PpIX-NLG@Lipo compared to PpIX@Lipo. This may give the credit to less PpIX-NLG aggregation in the hydrophobic layer or the solubility changes after conjugating with NLG919. Moreover, nearly no cytotoxicity was observed in PpIX-NLG@Lipo and PpIX@Lipo without LED light irradiation, which well demonstrated the spatiotemporal controllability of PDT, and laid the solid foundation to weaken the system toxicity when applied *in vivo*. Most importantly, whether with or without irradiation, the cell viability was not affected by NLG@Lipo at the concentration lower than 20 μM. Subsequently, the cytotoxicity of the liposomes was also confirmed visually by live/dead staining assay (**Figure 3C**). It could be seen obviously that there were almost live cells (green fluorescence) in the PBS control and NLG@Lipo treated group under irradiation. Moreover, the PpIX@Lipo and PpIX-NLG@Lipo treated cells without light irradiation also displayed almost green fluorescence, while obvious red fluorescence (dead cells) was exhibited after irradiation.

In order to further determine the mechanism of strong PDT efficacy induced by PpIX-NLG@Lipo, the Annexin V-PI analysis was applied. As illustrated in **Figure 3D**, significant amounts of cells underwent apoptosis/necrosis were occurred 4 h post the treatment of PDT induced by PpIX@Lipo and PpIX- NLG@Lipo, respectively, while a mass of number of cells were keep healthy after treatment with PBS or NLG@Lipo with irradiation. The difference results between Annexin V/PI assay and live/dead assay mentioned above may give the credit to the different further incubation time after LED light irradiation during the two experiments. In addition, those cells incubated with PpIX@Lipo or PpIX-NLG@Lipo without light irradiation showed a very high survival rate (>95%), which was in accordance with the results of live/dead cytotoxicity assay.

### CRT exposure and ATP secretion assay

**Figure 4:**
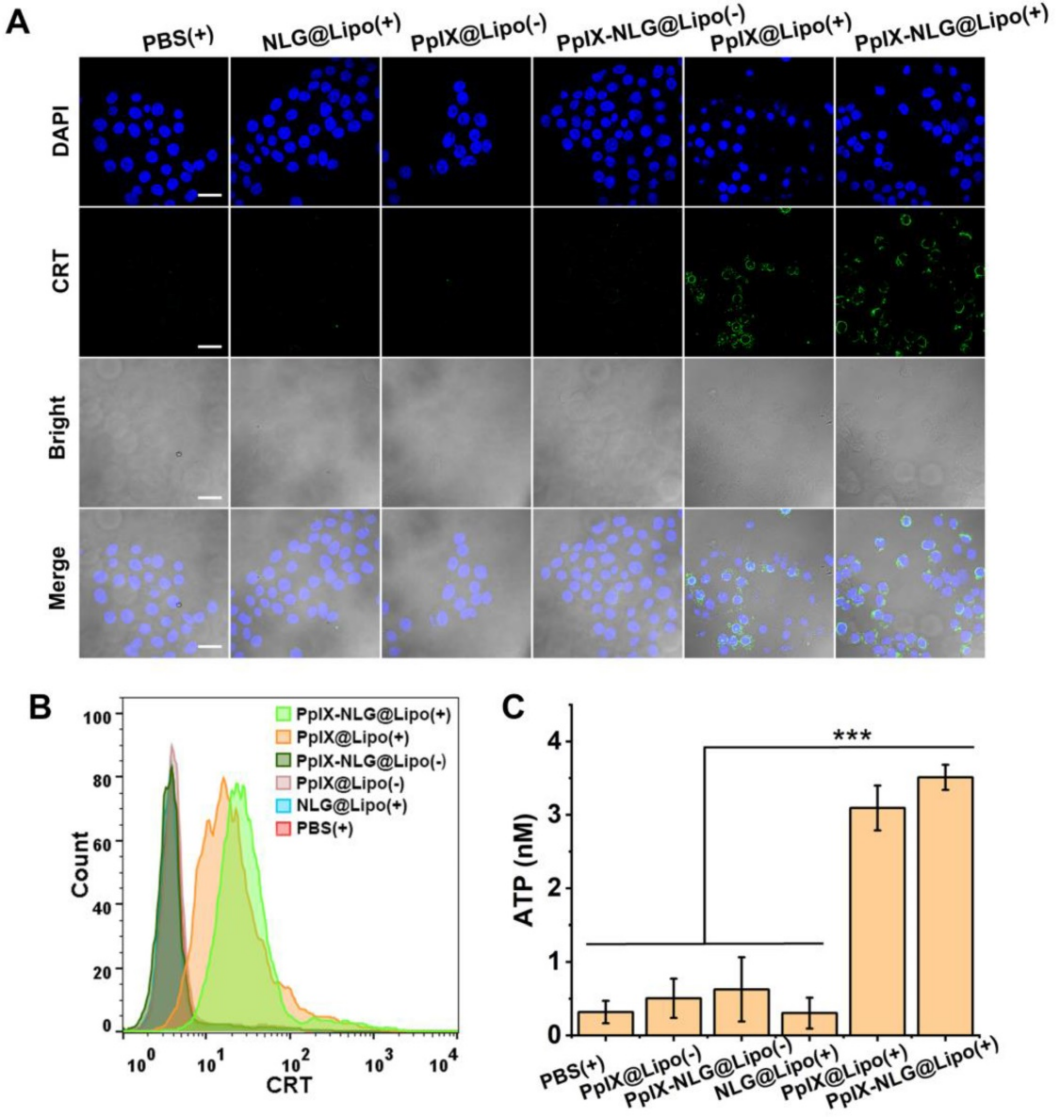
CRT expression of 4T1 cells with LED light irradiation (630 nm, 20 mW/cm^2^, 10 min) after incubation with PBS, NLG@Lipo, PpIX@Lipo or PpIX-NLG@Lipo, observed by CLSM (A) and quantified by FCM (B). (Scale bar = 20 μm). (C) Extracellular ATP release of 4T1 cells with LED light irradiation (630 nm, 20 mW/cm^2^, 10 min) after incubation with PBS, NLG@Lipo, PpIX@Lipo or PpIX-NLG@Lipo. (+) and (-) respectively refer to with or without irradiation.

Very recently, PDT was proved to cause immunogenically cell death (ICD) via inducing apoptosis and necrosis. During the ICD process, calreticulin (CRT), a chaperone protein abundant in the endoplasmic reticulum, was transported to the cell surface 32, which serves as an “eat me” signal, promoting the presentation of tumor-associated antigen to dendritic cells (DCs). In the meanwhile, ATP acts as “find me” signals regulate DC-mediated tumor antigen cross-presentation and T-cell polarization, finally activate the antitumor immune response 33. Thus, CRT exposure and ATP secretion were reported as the distinct biochemical hallmarks of ICD. Thus, after confirming the ability of inducing apoptosis or necrosis of PpIX-NLG@Lipo under light irradiation, the CRT exposure and ATP secretion was evaluated subsequently. To determine the CRT expression, after light irradiation, 4T1 cells were stained with Alexa Fluor 488-CRT antibody and DAPI, then observed by CLSM to visualize the CRT exposure (**Figure 4A**). Both PpIX@Lipo and PpIX-NLG@Lipo could induce CRT exposure under irradiation while PBS or NLG@Lipo showed little ability to induce CRT exposure. The FCM results (**Figure 4B**) was consistent with the CLSM results. As illustrated in **Figure 4C**, the supernatant ATP content was significantly enhanced after light irradiation treatments in the PpIX-NLG@Lipo incubation group compared to the dark treatments group as well as PBS control group. Taken together, PpIX-NLG@Lipo could induce CRT exposure and ATP secretion under light irradiation, indicating that PpIX-NLG@Lipo could apply as an ICD inducer to activate the host immune system under light irradiation. As a result, PpIX-NLG@Lipo was capable to induce ICD under light irradiation, laying a solid foundation for its further stimulate the host immune system.

### *In vitro* and *in vivo* IDO enzyme activity

**Figure 5:**
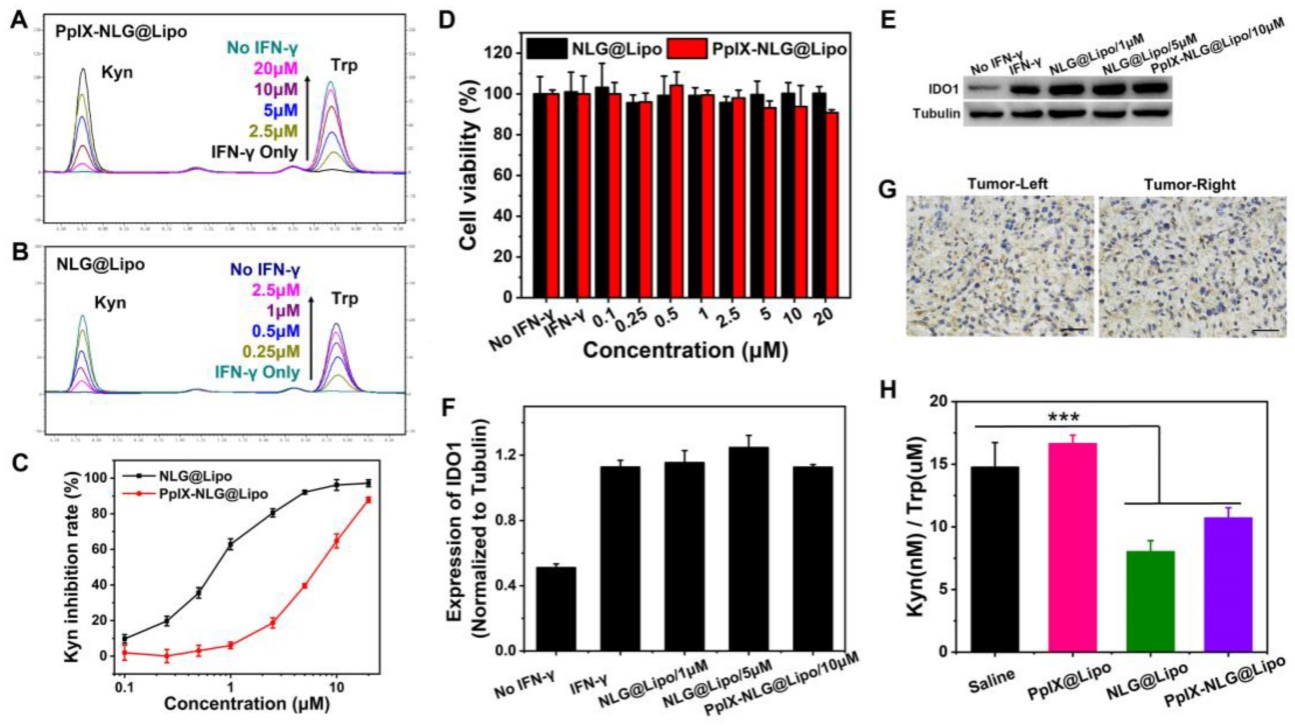
IDO enzyme activity analysis. After incubating with IFN-γ (50 ng/mL) and different concentration of PpIX-NLG@Lipo (A) or NLG@Lipo (B) for 48 h, the supernatants were entered to HPLC to detect the changes of Kyn and Trp. (C) Quantized Kyn inhibition rate of NLG@Lipo and PpIX-NLG@Lipo detected by colorimetric assay. (D) Cell viability of Hela cells treated with IFN-γ and NLG@Lipo or PpIX-NLG@Lipo for 48 h without light irradiation. (E, F) Western blot assay of IDO1 expression of Hela cells incubated with IFN-γ (50 ng/mL) and NLG@Lipo or PpIX-NLG@Lipo. (G) Representative images of immunohistochemistry staining of IDO antibody (positive expression of IDO shown in brown) of bilateral 4T1 tumor sections (Scale bar = 100 μm). (H) Kyn/Trp ratios in plasma of 4T1 tumor-bearing mice after different treatment.

Small-molecule IDO inhibitors, with the advantages of reversing tumor immunosuppressive microenvironment and inhibiting tumor growth, was widely developed by pharmaceuticals company all over the world 34. We hope that the introduction of IDO inhibitor can amplify PDT-mediated immune response, achieving both primary and distant tumor inhibition. Firstly, to evaluate the IDO enzyme activity inhibition of PpIX-NLG@Lipo, IFN-γ was applied to promote IDO expression of Hela cells 35. From western blot assay (**Figure 5E**), the IDO expressed significantly more in Hela cells after IFN-γ pre-treating. Moreover, from HPLC chromatogram of PBS group and IFN-γ treated group (**Figure 5A**, **Figure 5B**), Trp in supernatants can transform into Kyn completely under IDO catalysis. That is, IFN-γ can stimulate higher IDO expression, the IDO activity inhibition can be characterized by inhibiting the conversion of Trp to Kyn under IFN-γ stimulation.

From **Figure 5A**, when IFN-γ was incubated together with PpIX-NLG@Lipo, Trp cannot be oxidized to Kyn fully under catalysis of IDO, furthermore, with the concentration of PpIX-NLG@Lipo increasing from 2.5 μM to 20 μM, Kyn decreasing as well as Trp increasing at the same time, suggesting that PpIX-NLG can weaken the enzyme activity of IDO so that to inhibit the conversion of Trp to Kyn. The capacity of IDO activity inhibition of NLG@Lipo was also examined in the meanwhile, in **Figure 5B** and **Figure 5C**, NLG@Lipo showed an excellent ability of Kyn inhibition under the low concentration (0.25 μM to 2.5 μM), with an effective concentration (EC_50_) of 0.7 μM. Whereas, PpIX-NLG@Lipo inhibited 50% of IDO activity at an effective concentration of 6 μM, 8.5-folds lower than those of NLG@Lipo, which could most likely to be explained by the declined affinity between PpIX-NLG and IDO after conjugating PpIX to NLG919.

From western blot assay, the IDO expression in Hela cells did not change significantly after co-incubation with drug and IFN-γ, indicating that the mechanism of NLG919 or PpIX-NLG decreasing kyn was not inhibit the IDO expression but the activity of IDO. In addition, the cell viability of Hela cells after co-treating with prepared liposomes and IFN-γ was also determined at the same time (**Figure 5D**). Hela cells from both two treatment groups still live during the experiment, suggesting that IFN-γ showed little cytotoxicity and the results of IDO activity assay was not influenced by the cell death. The mentioned above results demonstrated that PpIX-NLG@Lipo and NLG@Lipo was capable to inhibit IDO enzyme activity under the premise that did not affect the IDO expression as well as the cell viability.

In this study, the high expression of IDO in the tumor microenvironment was the precondition for effective of IDO inhibitors. Thus, immunohistochemistry (IHC) was applied to detect the IDO expression in the tumor site of bilateral 4T1 mouse breast cancer model mice. After the bilateral 4T1 mouse breast cancer model have been established, some of mice was sacrificed and removed their tumors, then IHC staining of those tumor sections was performed with the aim to determine the IDO expression in tumor tissues. As depicted in **Figure 5G**, the brown region was seen both in the left and right tumor, which revealed the IDO positive expression in the established bilateral 4T1 mouse breast cancer model 36.

The potency of inhibiting IDO activity by PpIX-NLG@Lipo *in vivo* was evaluated by examining the Kyn/Trp ratios in the plasma of 4T1 tumor- bearing mice after administration. From** Figure 5H**, comparing with the saline and PpIX@Lipo group, the Kyn(nM)/Trp(μM) ratios was reduced following the treatment of NLG@Lipo and PpIX-NLG@Lipo, in accordance with their *in vitro* results of IDO activity inhibition experiment. Besides, compared with PpIX-NLG@Lipo group, a small reduction was also observed in NLG@Lipo group, that is, the ability difference between NLG@Lipo and PpIX-NLG@Lipo in inhibition of IDO activity *in vivo* seems smaller than that of *in vitro*, maybe due to the fact that PpIX-NLG can partly transformed back to NLG919 under the effect of esterase *in vivo* environment.

### *In vivo* tumor imaging and pharmacokinetics study

**Figure 6:**
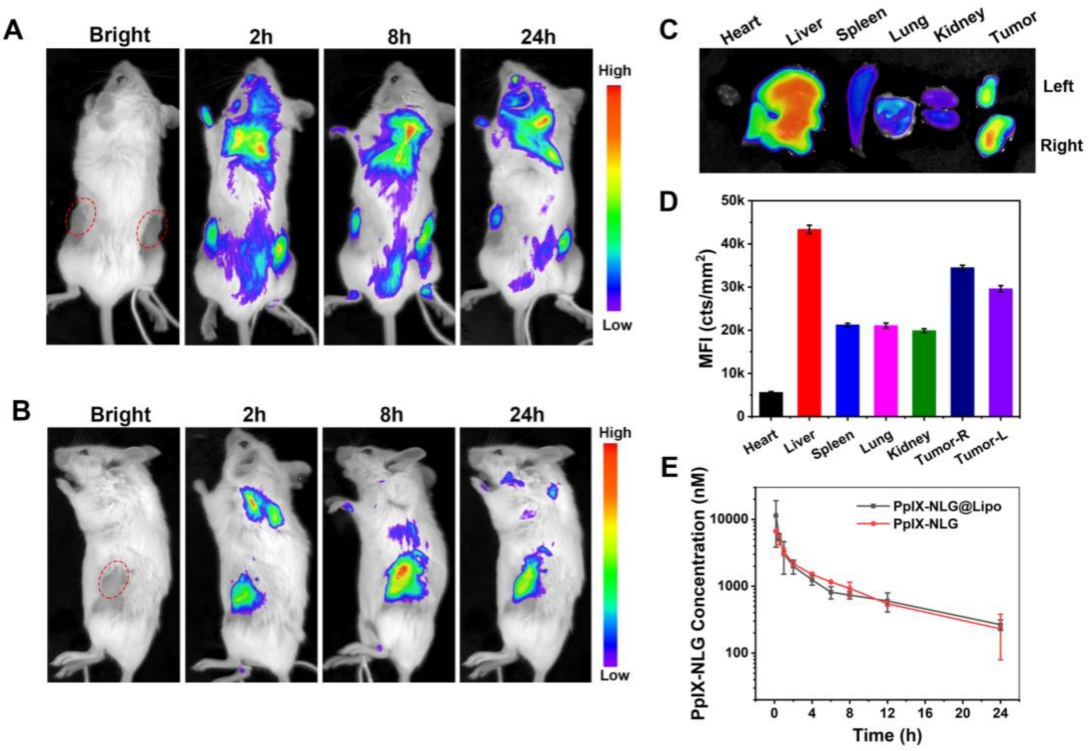
Representative *in vivo* fluorescence imaging of bilateral 4T1 tumor-bearing mice at the time points of 2 h, 8 h, and 24 h post-intravenous injection of PpIX-NLG@Lipo, the prone position view (A) and side-lying position view (B). The ex vivo fluorescence imaging (C) and mean fluorescence intensity (MFI) values (D) of the major organs and tumors at 24 h post-injection. (E) PpIX-NLG concentration of plasma of healthy rats after I.V. injection of PpIX-NLG@Lipo or free PpIX-NLG.

The specific accumulation of PpIX-NLG@Lipo in the tumor site was very fundamental to the treatment efficiency *in vivo* application. With the advantage of fluorescence of PpIX-NLG@Lipo, the drug distribution can be noninvasively tracked. Thus, we examined the specific distribution in tumor tissues of the bilateral 4T1 tumor-bearing mice after intravenous injection of PpIX-NLG@Lipo by using an animal imaging system. As shown in** Figure 6A**, fluorescence was observed both in the left and right tumor site after 2 h post-injection, and gradually enhanced till 8 h. It can be seen more clearly from the side-lying position view that the fluorescence intensity in the tumor region reached to a max value at the time point of 8 h (**Figure 6B**), furthermore, the accumulation fluorescence intensity in the tumor tissue was still obviously strong even after 24 h post-injection (**Figure 6C**, **Figure 6D**), suggesting that PpIX-NLG@Lipo show a high accumulation in tumor site, which laid a solid foundation for its *in vivo* application. The in vivo pharmacokinetics study was determined in rats by tail vein injection of PpIX-NLG@Lipo or PpIX-NLG (which was dissolved in saline containing 3% DMSO and 3% tween-80). As illustrated in **Figure 6E**, PpIX-NLG@Lipo and free PpIX-NLG shown the similar blood circulation time. The reason for this result was that the liposome was prepared only with DOPC and cholesterol while without a PEG shell. In addition, tween-80 was applied to solubilize the free PpIX-NLG, leading to a micelle of PpIX-NLG, which may cause the similar pharmacokinetics property between PpIX-NLG@Lipo and free PpIX-NLG.

### *In vivo* antitumor effect and mechanisms analysis

Considering the promising results of PpIX-NLG @Lipo in tumor accumulation and IDO activity inhibition *in vivo*, the abscopal antitumor activities in the bilateral 4T1 murine breast cancer model was applied to evaluate the synergistic therapeutic effect of PDT and IDO blockade. A bilateral syngeneic mouse model was employed in this experiment, in which 4T1 cells were s.c. inoculated into both the left and right flanks of BALB/c mice. The right-side tumor as primary tumor was designed for laser irradiation to induce PDT treatment, while the tumors on the left side were designated to the distant tumors without laser irradiation.

**Figure 7:**
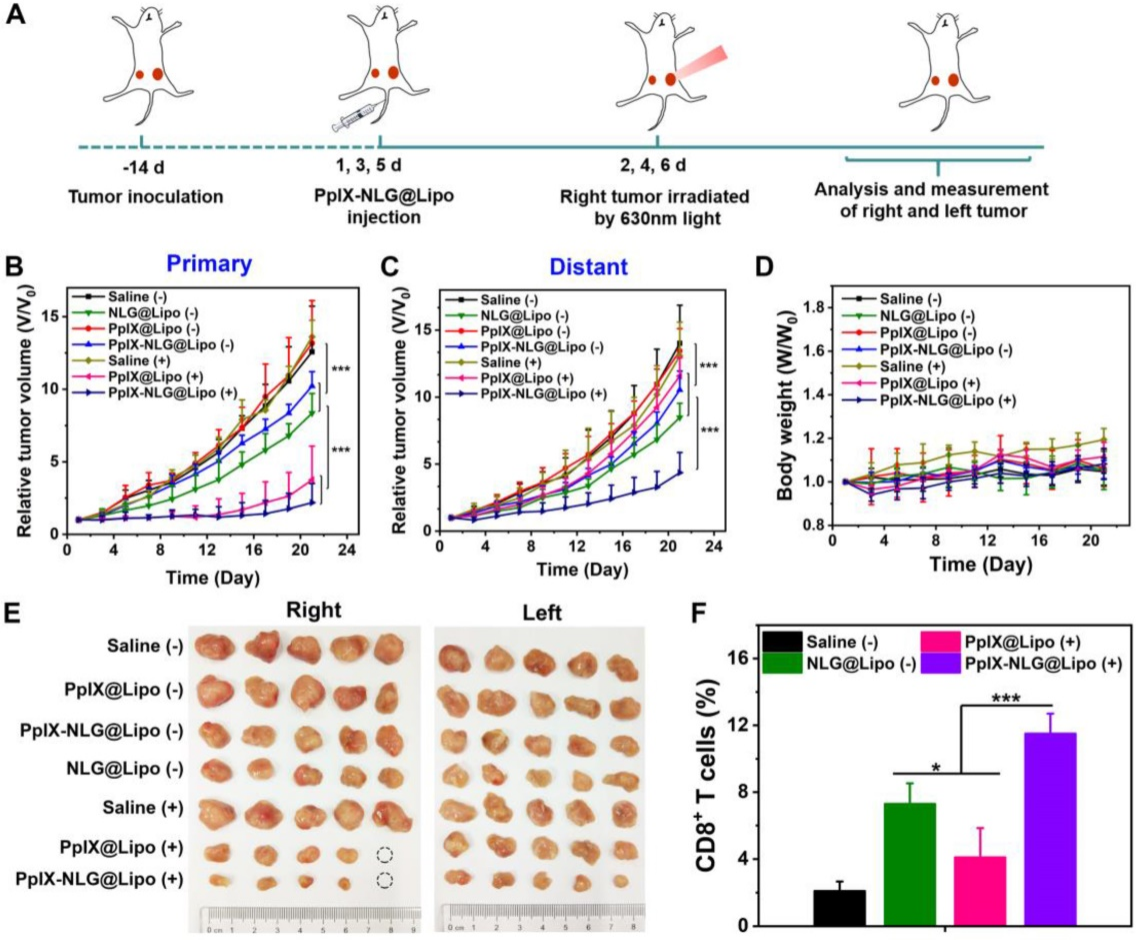
The abscopal antitumor effect of PpIX-NLG@Lipo in the bilateral 4T1 tumor-bearing mice. (A) Schematic illustration of the experiment design. Mice with 4T1 tumor in both sides were used in this experiment, and tumors on the right side were designated to the “primary tumors” for light-mediated PDT treatment, while the tumors on the left side were designated to the “distant tumors” without light irradiation. Primary (B) and distant (C) tumor growth curves of mice after various treatments. Body weight curves (D) of mice of different group. (E) Photos of excised primary (right) and distant (left) tumors from each treatment group. (F) Proportion of CD8^+^ T cells infiltration in the distant tumors detected by FCM.

When the primary tumor reached 80~100mm^3^, as described in **Figure 7A**, mice received systemically administration with different formulations for a total of three injections, followed by laser irradiation (630 nm, 300 mW/cm^2^, 10 min) on primary tumors. As shown in **Figure 7B**, the group of saline with laser irradiation and PpIX@Lipo without laser irradiation all showed negligible inhibition in tumor volume of primary tumor, suggesting that pure laser as well as PpIX@Lipo without laser irradiation had no obvious effect on tumor growth. Moreover, administration of NLG@Lipo or PpIX-NLG@Lipo without laser irradiation could suppress the tumor growth in primary tumor due to the inhibition of IDO activity, indicating that antitumor immune response could be roused to some extent after IDO activity inhibition. However, NLG@Lipo exhibited better therapeutic effect on inhibiting tumor growth than that of PpIX-NLG@Lipo, which was consistent with the outcomes of IDO activity inhibition experiment *in vivo*. Both PDT induced by PpIX@Lipo and PpIX-NLG@Lipo under a laser irradiation could excellently inhibit tumor growth to a very large degree (**Figure 7E**), furthermore, after the treatment of PpIX-NLG@Lipo, the tumor growth rate was slower than that of PpIX@Lipo in the later stage, mainly due to the synergistic effect of IDO activity inhibition.

The aim of bilateral tumor model was to evaluate therapeutic outcome of PpIX-NLG@Lipo to inhibit primary and distant metastatic tumors in the meanwhile. As shown in **Figure 7C** and **Figure 7E**, compared to the saline group, the pure laser as well as PpIX@Lipo without laser irradiation could not inhibit tumor growth obviously, while NLG@Lipo and PpIX-NLG@Lipo without laser irradiation group showed a slower tumor growth because of the immune response induced by IDO activity inhibition, which was in accordance with that of primary tumor. Interestingly, PDT induced by PpIX@Lipo in primary tumor could inhibit the growth of distant tumor at a short of the beginning, but the increasing rate of distant tumor volume became faster at the later stage of the experiment. In sharp contrast, a significant inhibition of growth of distant tumor was observed in the PpIX-NLG@Lipo treated group, suggesting that synergetic therapy of PDT and IDO activity inhibition displayed increased antitumor effect compared to monotherapy.

To elucidate the mechanisms underlying the distant tumor inhibition efficacy of PpIX-NLG@Lipo under laser irradiation, we examined the infiltration of CD8^+^ T cells in the distant tumor site after a total three treatments of PpIX-NLG@Lipo by FCM and IHC. As displayed in **Figure 7F** and **Figure S6**, compared to the saline control group, more CD8^+^ T cells were generated in distant tumor after PpIX@Lipo induced PDT treatment, which may the reason for the inhibition of distant tumor at the short beginning. Furthermore, there were more CD8^+^ T cells observed in NLG@Lipo group, which could in accordance with the results of *in vivo* IDO activity inhibition assay, suggesting that NLG919 could block IDO activity to decrease Kyn/Trp ratio *in vivo*, and then induce increasing CD8^+^ T cells infiltration into tumor site to achieve antitumor immunotherapy. Most interestingly, a large amount of CD8^+^ T cells were infiltrated into tumor site after PpIX-NLG@Lipo mediated PDT treatment, which was more than that of PpIX@Lipo induced PDT treatment as well as NLG@Lipo mediated IDO blockade treatment. This demonstrated that PpIX-NLG@Lipo mediated PDT could stimulate the host immune system to induce a small amount of CD8^+^ T cells production, and then its ability of IDO activity blockade could strengthen the immune response caused by PDT, resulting in a robust immune response, which may achieve enhanced distant tumor inhibition.

From what was discussed above, PpIX-NLG@ Lipo showed obvious therapeutic effect on inhibiting primary tumor and distant tumor to some extent. Subsequently, the infiltration of CD8^+^ T cells into distant tumor site was detected to explain the abscopal anti-tumor effect of PpIX-NLG@Lipo. However, the role of other immune related factors and other immunocytes, such as NK cells, CD4^+^T cells, Treg cells were not evaluated together to elucidate the antitumor immune response of PpIX-NLG@Lipo, which we are going to make efforts next.

### Safety evaluation

**Figure 8:**
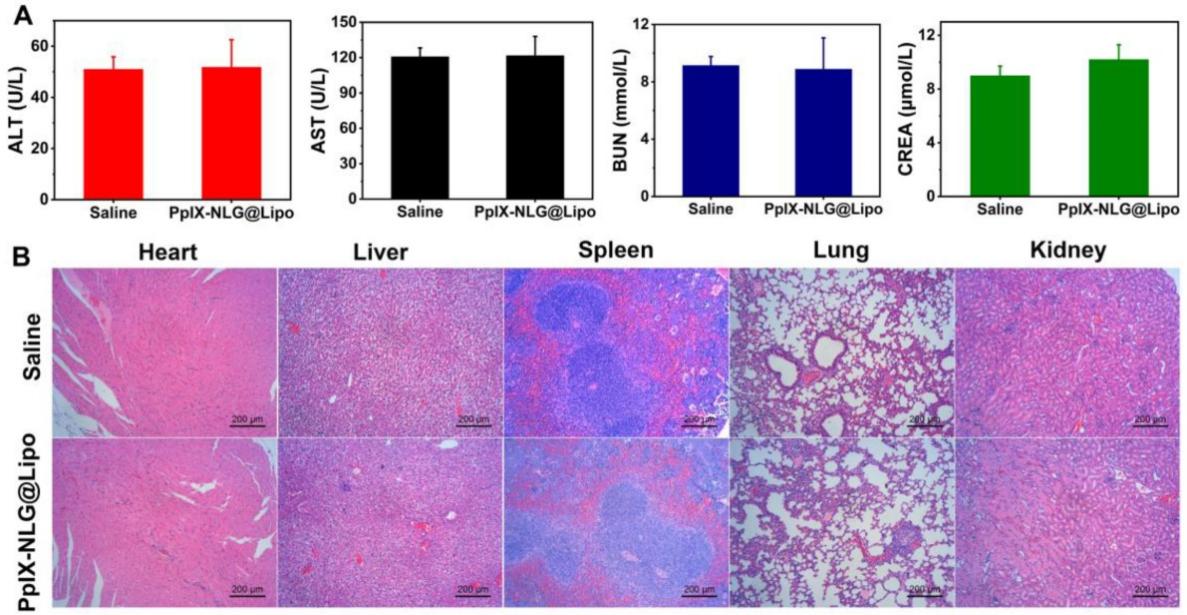
Evaluation of the systemic safety of PpIX-NLG@Lipo. (A) Levels of blood biochemical indexes: ALT, AST, BUN and CREA, after single intravenous injection of saline or PpIX-NLG@Lipo. (B) Histopathology of heart, liver, spleen, lung and kidney after intravenous injection of saline or PpIX-NLG@Lipo.

To evaluate the system toxicity of PpIX-NLG@ Lipo *in vivo* application, blood biochemical indexes and histopathology of the vital organs were determined. ALT and AST were measured to evaluate the live function, while BUN and CREA were tested to estimate renal function. As shown in** Figure 8A**, after a single intravenous injection of PpIX-NLG@Lipo, the above blood biochemical indexes exhibited insignificant changes compared to the saline control group, suggesting that the systematical administration of PpIX-NLG@Lipo showed negligible toxicity of liver and kidney. This was further confirmed by observing the histopathology of the heart, liver, spleen, lung and kidney of mice treated with PpIX-NLG@Lipo after stained with H&E (**Figure 8B**). Compared to the saline group, there were negligible pathological changes on the vital organs after treated with PpIX-NLG@Lipo, indicating the good security of single administration of PpIX-NLG@Lipo. Furthermore, the body weight changes during the *in vivo* antitumor experiment is also the indicator of the system toxicity (**Figure 7D**), which also suggested inappreciable toxicity of PpIX-NLG@Lipo during PDT.

## Conclusion

In summary, we developed a liposomal dual functional conjugate comprised of PpIX and NLG919 for synthetic PDT and IDO blockade to inhibit primary and distant metastatic tumor at the same time. The as-prepared liposome PpIX-NLG@Lipo exhibited a uniform size distribution at the mean diameter of 100 nm, showed a high accumulation in tumor site, laying a solid foundation for the *in vivo* application. Meanwhile, the fluorescence of PpIX-NLG@Lipo allowed noninvasive tracking of drug distribution. The vitro cytotoxicity experiment demonstrated that the negligible phototoxicity, good ROS generation and effective PDT therapy of PpIX-NLG@Lipo. Moreover, the *in vitro* cell experiment indicated that PpIX-NLG@Lipo could cause ICD via apoptosis and necrosis during PDT treatment, which may present tumor-associated antigens to stimulate the host immune system. Furthermore, PpIX-NLG@Lipo could blockade IDO function by inhibiting its activity to converse Trp to Kyn both *in vitro* and *in vivo*. The antitumor experiment on bilateral 4T1 murine breast cancer model demonstrated that PpIX-NLG@Lipo mediated PDT could damage tumor cells directly to inhibit the growth of primary tumor, and simultaneously present tumor-associated antigens to stimulate the host immune system, then PpIX-NLG could inhibit IDO pathway to achieve effectively distant tumor inhibition. Therefore, such a PpIX-NLG@Lipo exhibited a promising application in synergetic PDT and IDO blockade for enhanced cancer therapy. Furthermore, such a treatment strategy that combining PDT and IDO blockade provides great potential for enhanced cancer therapy through simultaneously inhibiting both primary and distant metastatic tumor.

## Abbreviations

IDO: indoleamine-2,3-dioxygenase
PpIX: protoporphyrin Ⅸ
PDT: photodynamic therapy
ROS: reactive oxygen species
ICD: immunogenic cell death
CD8^+^T cells: CD8^+^ T lymphocytes
Trp: tryptophan
Kyn: kynurenine
ABDA: 9,10-anthracenediyl-bis(methylene)dimalonic acid
FDA: fluorescein diacetate (FDA)
DCFH-DA: 2′,7′-dichlorofluorescein diacetate
MTT: 3-(4.5-dimethyl-thiazol-2-yl)-2.5-diphenyl tetrazolium bromide
DPBF: 1,3-Diphenylisobenzofuran
PI: propidium iodide
DOPC: 1,2-dioleoylsn-glycero-3-phosphocholine
DMEM: Dulbecco's Modified Eagle's Medium
FBS: fetal bovine serum
IHC: immunohistochemistry
DCs: dendritic cells
CRT: calreticulin (CRT)
ALT: alanine aminotransferase
AST: aspartate aminotransferase
BUN: urea nitrogen
CREA: creatinine
