# Supplementary material for: Enhanced cancer therapy through synergetic photodynamic/immune checkpoint blockade mediated by a liposomal conjugate comprised of porphyrin and IDO inhibitor
Source: Theranostics. 2019 Jul 29;9(19):5542–57. doi: 10.7150/thno.35343 (PMC6735384; doi:10.7150/thno.35343)
Supplement: Supplementary file 1 — Supplementary figures. [file thnov09p5542s1.pdf]

Supporting information

Enhanced cancer therapy through synergetic photodynamic/immune checkpoint blockade mediated by a liposomal conjugate comprised of porphyrin and IDO inhibitor

Zeqian Huang[1], Gaofei Wei[1], Zishan Zeng, Yanjuan Huang, Liangfeng Huang, Yifeng Shen, Xiaoqi Sun, Congjun Xu, Chunshun Zhao*

School of Pharmaceutical Sciences, Sun Yat-sen University, Guangzhou 510006, People's Republic of China

* Corresponding author:

Chunshun Zhao. E-mail address: zhaocs@mail.sysu.edu.cn

[1] These authors contributed equally to this work.

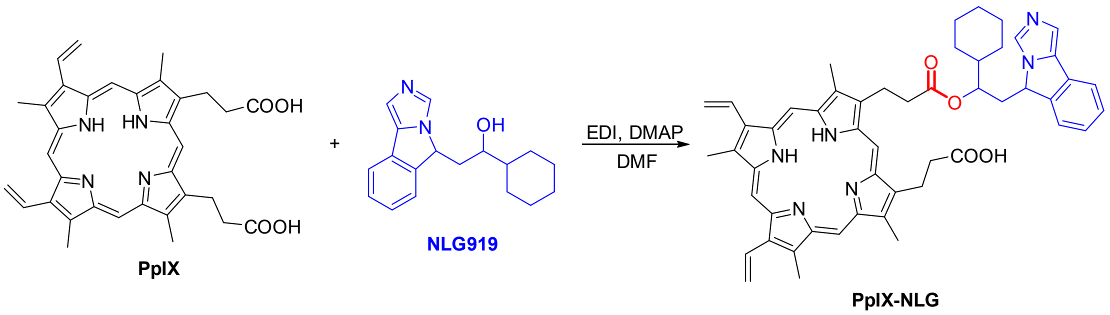

Scheme S1. Synthesis of the conjugate PpIX-NLG.

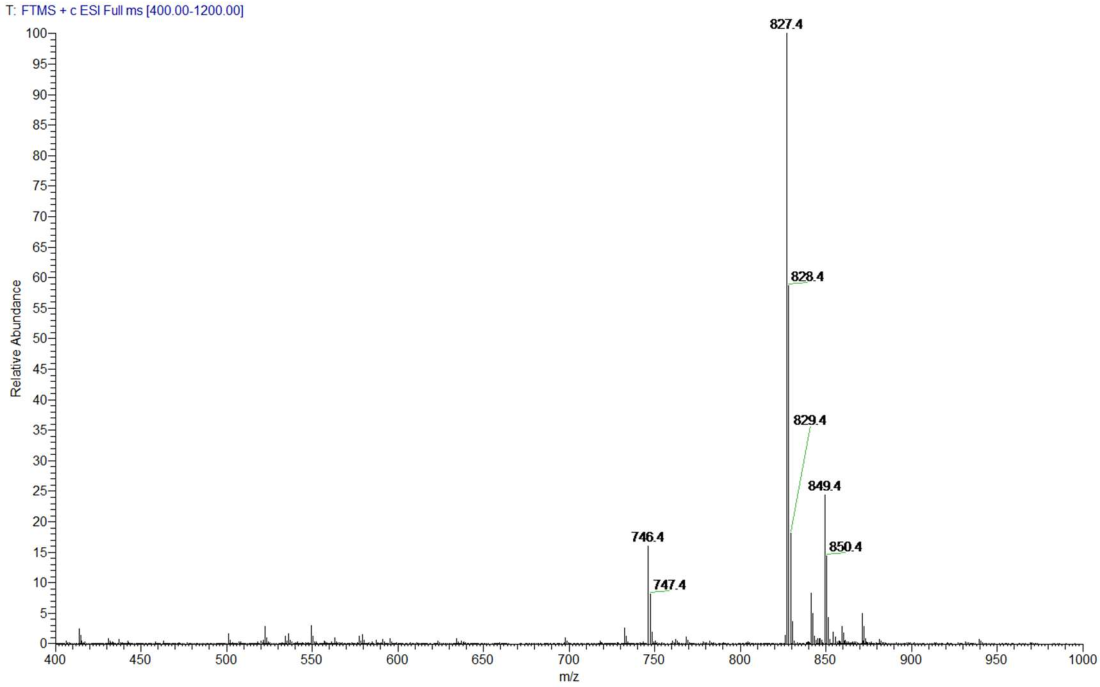

Figure S1. ESI - High resolution mass spectra (HRMS) of PpIX-NLG.

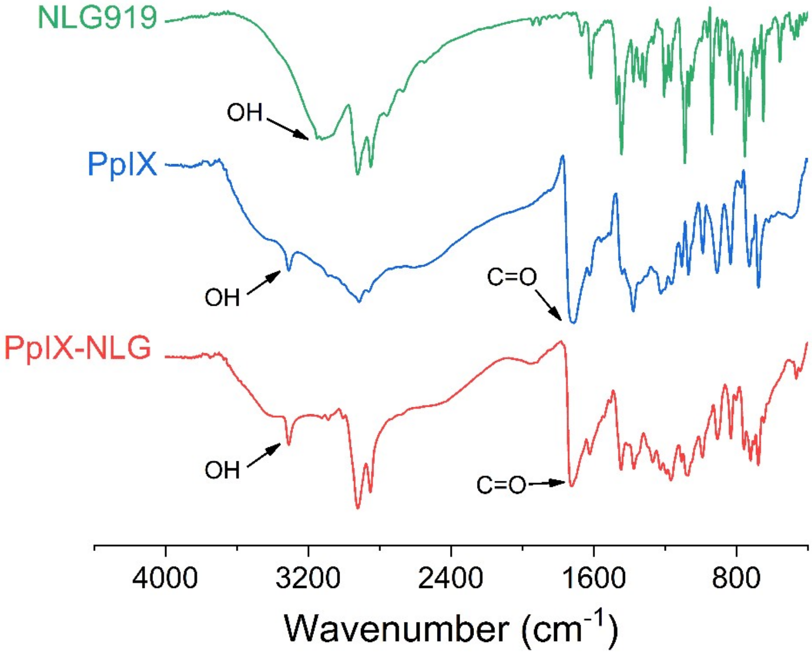

Figure S2. FTIR spectra of PpIX, NLG919 and PpIX-NLG.

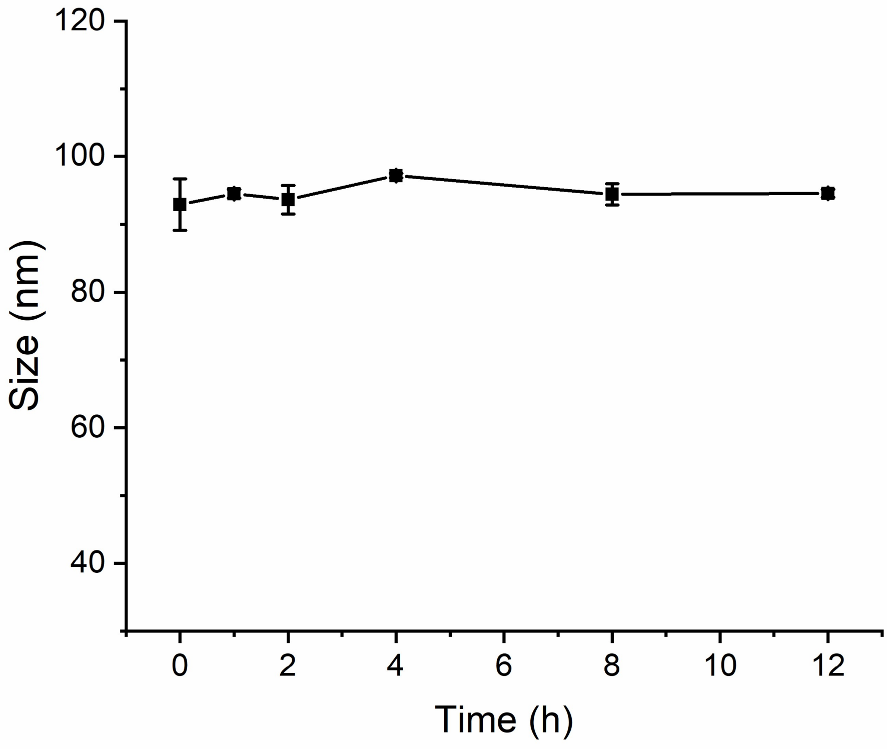

Figure S3. Stability of PpIX-NLG@Lipo in PBS buffer with 10% FBS at 37℃.

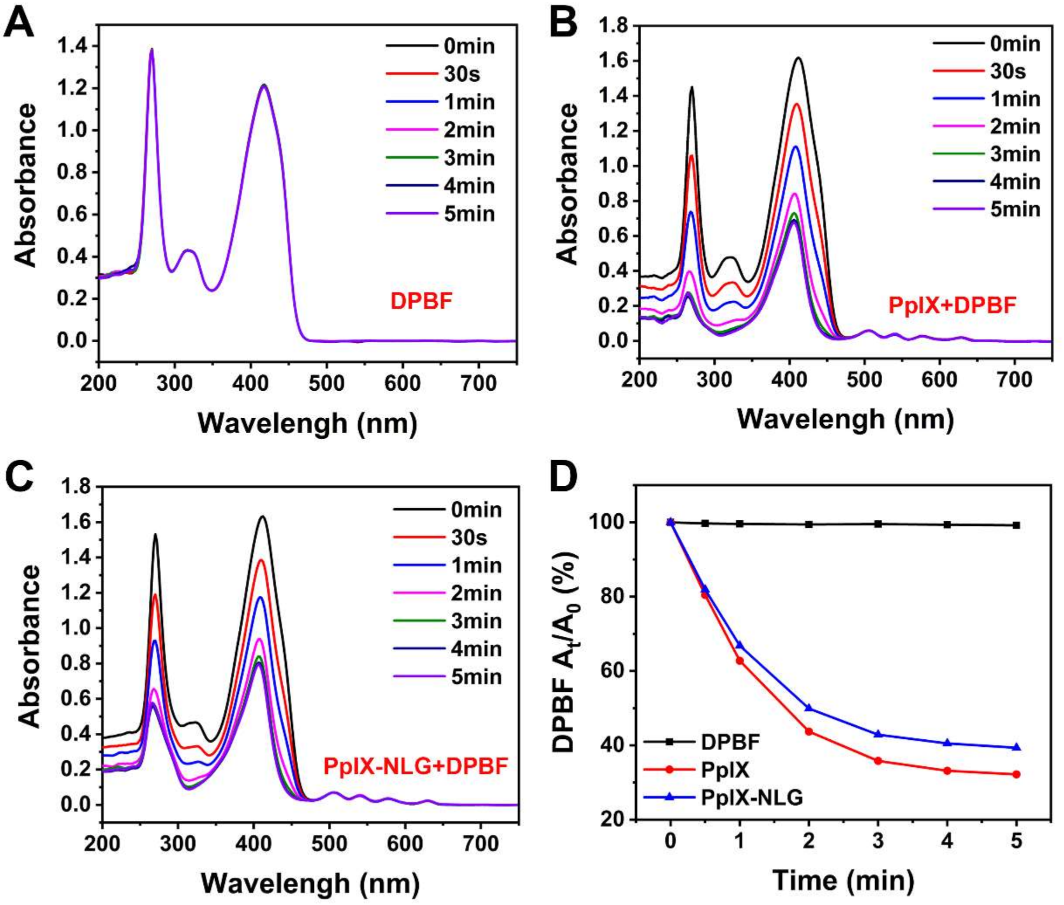

Figure S4. The detection of ROS generation using DPBF as ROS indicator in DMSO. The UV-vis spectra changes of DPBF (A), DPBF and PpIX mixture (B), DPBF and PpIX-NLG mixture (C) at different time under LED light irradiation (630 nm, 20 mW/cm^2). (D) The decrement curve of absorbance at 416 nm of DPBF, PpIX and PpIX-NLG under LED light irradiation (630 nm, 20 mW/cm^2).

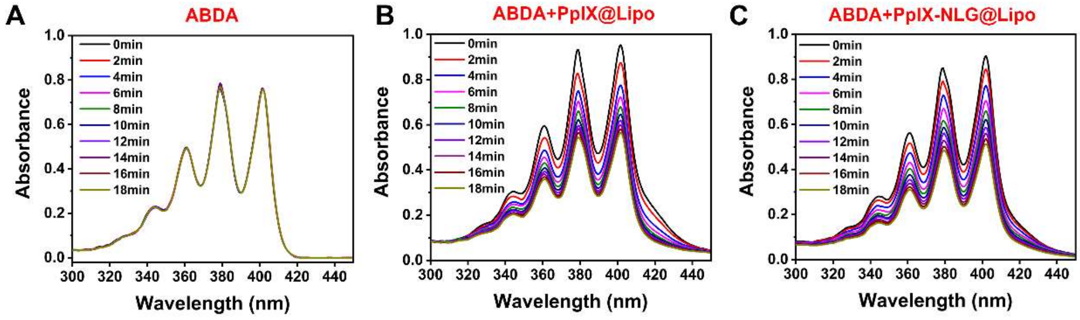

Figure S5. The detection of ROS generation from the prepared liposomes using ABDA as ROS indicator in the aqueous solution. The UV-vis spectra changes of ABDA (A), ABDA and PpIX@Lipo mixture (B), ABDA and PpIX-NLG@Lipo mixture (C) at different time under laser irradiation (630 nm, 50 mW/cm^2).

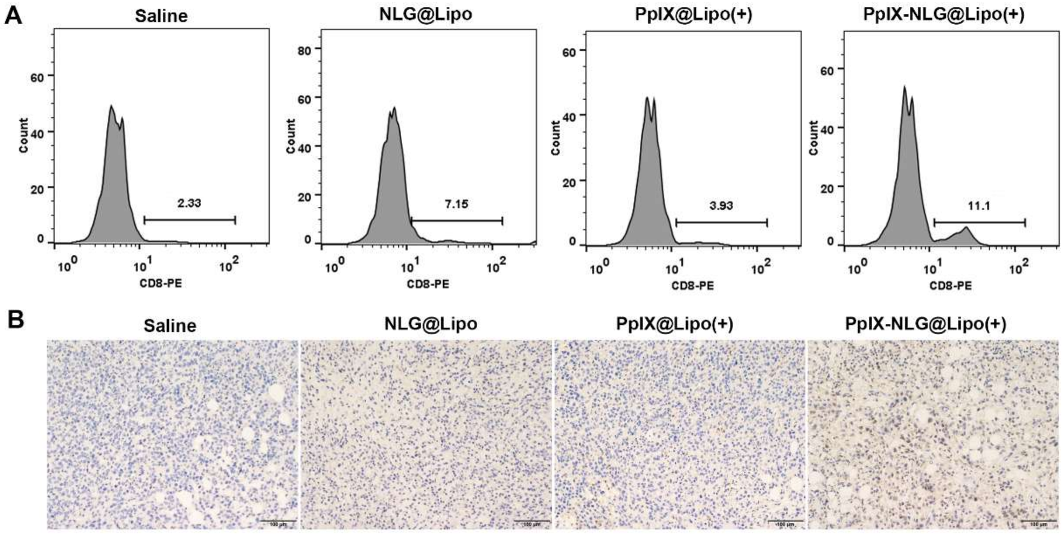

Figure S6. After different treatments in the bilateral 4T1 tumor-bearing mice, distant tumor was removed, $CD8^+$ T cells infiltration in the distant tumors detected by FCM (A) and IHC (B) to determine the immune response in vivo.
